# A novel similarity-measure for the analysis of genetic data in complex phenotypes

**DOI:** 10.1186/1471-2105-10-S6-S24

**Published:** 2009-06-16

**Authors:** Vincenzo Lagani, Alberto Montesanto, Fausta Di Cianni, Victor Moreno, Stefano Landi, Domenico Conforti, Giuseppina Rose, Giuseppe Passarino

**Affiliations:** 1 Department of Electronis, Informatics and Systems, University of Calabria, Via Ponte Pietro Bucci 41C, 87036, Rende, Italy; 2 CEntro di Supercalcolo per l'Ingegneria Computazionale, University of Calabria, Via Ponte Pietro Bucci 21B, 87036 Rende Italy; 3 Department of Cell Biology, University of Calabria, Via Ponte Pietro Bucci 4C, 87036, Rende, Italy; 4 Biostatistics and Bioinformatics Unit, IDIBELL-Catalan Institute of Oncology, Gran Via km 2.7, 08907 L'Hospitalet del Llobregat, Barcelona, Spain; 5 Department of Biology, University of Pisa, Via Derna 1, 5216, Pisa, Italy

## Abstract

**Background:**

Recent technological advances in DNA sequencing and genotyping have led to the accumulation of a remarkable quantity of data on genetic polymorphisms. However, the development of new statistical and computational tools for effective processing of these data has not been equally as fast. In particular, Machine Learning literature is limited to relatively few papers which are focused on the development and application of data mining methods for the analysis of genetic variability. On the other hand, these papers apply to genetic data procedures which had been developed for a different kind of analysis and do not take into account the peculiarities of population genetics. The aim of our study was to define a new similarity measure, specifically conceived for measuring the similarity between the genetic profiles of two groups of subjects (i.e., cases and controls) taking into account that genetic profiles are usually distributed in a population group according to the Hardy Weinberg equilibrium.

**Results:**

We set up a new kernel function consisting of a similarity measure between groups of subjects genotyped for numerous genetic loci. This measure weighs different genetic profiles according to the estimates of gene frequencies at Hardy-Weinberg equilibrium in the population. We named this function the "Hardy-Weinberg kernel".

The effectiveness of the Hardy-Weinberg kernel was compared to the performance of the well established linear kernel. We found that the Hardy-Weinberg kernel significantly outperformed the linear kernel in a number of experiments where we used either simulated data or real data.

**Conclusion:**

The "Hardy-Weinberg kernel" reported here represents one of the first attempts at incorporating genetic knowledge into the definition of a kernel function designed for the analysis of genetic data. We show that the best performance of the "Hardy-Weinberg kernel" is observed when rare genotypes have different frequencies in cases and controls. The ability to capture the effect of rare genotypes on phenotypic traits might be a very important and useful feature, as most of the current statistical tools loose most of their statistical power when rare genotypes are involved in the susceptibility to the trait under study.

## Background

Recent advances in DNA technology have led to the accumulation of a remarkable quantity of data on genetic polymorphisms. Consequently, there has been a growing interest in the possibility of carrying out studies for a variety of human complex traits, that is traits due to the variability of many genes, each contributing a minor or very small effect. For instance, many studies have been devoted to the analysis of the genetic components of cancer, cardiovascular diseases, dementias, and aging. In particular, the availability of ultra-high-volume genotyping platforms at a manageable cost has permitted genome-wide association studies where genetic profiles observed in groups of affected subjects (cases) are compared to groups of healthy subjects (controls) in order to identify multiple low-penetrance variants involved in complex phenotypes [1-5]. In fact, the number of genome-wide association studies aimed to identify genetic variants involved in complex phenotypes has recently increased exponentially [6-11]. However, the development of new statistical and computer based tools for the effective processing of the large amount of data arising from these studies has not evolved equally as fast (for a review see [12]).

Recently, kernel-based methods have attracted the attention of many researchers in the broad field of Knowledge Discovery and Machine Learning methodologies. The main aim of a kernel-based method is to devise a suitable kernel function to encode a similarity among the entities of the data set. In this framework many kernel-based methods have been developed, e.g. kernel principal component analysis [13], kernel logistic regression [14], and kernel Fisher discriminant analysis [15]. More specifically, kernel-based methods have become popular tools in the Machine Learning community since the introduction of Support Vector Machines (SVMs) during the early 1990s [16].

SVM represent a set of data mining methods that, taking advantage of a kernel function, are used to analyze large datasets in order to perform classification, clustering, and regression analysis [17]. For instance, they are widely used to analyze very large sets of gene-expression data. However, different studies have shown that the performance of a SVM classifier is strongly related to the similarity measure used in the classifier (kernel function) [16,17]. In spite of their well known statistical power, to date Machine Learning literature relies on relatively few papers which focus on the development and application of data mining methods specifically devised for the analysis of genetic polymorphisms [18-23]. However, this field lacks the development of specific measures that take into account the main laws regulating the dynamics of population genetics.

The aim of our study was to define a new similarity measure specifically conceived for incorporating the knowledge of population genetics into the study of genetic datasets obtained from high throughput analysis for association studies. The main feature of such measure is that the similarity between groups of subjects typed for their genetic profiles is weighed according to the estimates of gene frequencies at Hardy-Weinberg equilibrium in the population. The Hardy-Weinberg equilibrium represents the main principle regulating population genetics. It states that allele and genotype frequencies in a population are constant – that is, they are in equilibrium- from generation to generation unless specific disturbing influences are introduced. This allows the estimation of the expected frequency of a genotype on the basis of allele frequencies and *vice versa*. Thus, we named this kernel the "Hardy-Weinberg kernel" (HWk). This kernel, estimates the similarity between different genetic profiles based on the frequency of each genotype in the general population. Consequently, once embedded in a SVM classifier, HWk allows to estimate the influence of each genotype on the probability of it being part of a given group of subjects (for instance a group of subjects with a given phenotype or disease).

## Methods

### Data encoding

In the present study, a *dataset *is a table composed of *n *rows, namely *X*_1_, *X*_2_,..., *X*_*n*_, where *X*_*i *_represents the genetic profile of a single subject. The subjects typed for their genetic profiles are subdivided into two different classes, i.e. class +1 and -1, reflecting the presence or absence of one specific phenotype (i.e. subjects belonging to class +1 are the "cases" and -1 are the "controls"). Each row *X*_*i *_is composed of *m *variables, *SNP*_1_, *SNP*_2_,..., *SNP*_*m*_, representing the set of Single Nucleotide Polymorphisms (SNPs) under study (see Table 1). Lastly, each variable *SNP*_*j *_can assume one of three values, {*A*_1_*A*_1_, *A*_1_*A*_2_, *A*_2_*A*_2_}, each value corresponding to one combination (i.e. genotype) of the generic alleles *A*_1 _and *A*_2 _(*A*_1_*A*_2 _∈ *A*_2_*A*_1_).

**Table 1:** Example of a genetic dataset with two SNPs

ID	SNP_1_	SNP_2_	Class
SAMPLE1	A_1_A_1_	A_1_A_1_	+1
SAMPLE2	A_2_A_2_	A_1_A_2_	-1
SAMPLE3	A_1_A_2_	A_2_A_2_	-1

Since standard SVM classifiers cannot handle categorical variables directly, SNPs data must be encoded in a numerical format. A possible solution consists in constructing three binary variables, *SNP*_*jk *_(k = 1, 2, 3), for each *SNP*_*j*_; in this way each binary variable represents one of the three possible genotypes of the relevant SNP. By encoding in numerical format, each row *X*_*i *_of the *dataset *will contain 3· *m *binary variables (Table 2); the generic element of the row *X*_*i *_will be indicated as , i.e.,  takes value *1 *if the *i*^*th *^genetic profile present the genotype *k *for the SNP *j*, and value *0 *otherwise (Table 2).

**Table 2:** Numeric encoding of Table 1

ID	SNP_11_ (A_1_A_1_)	SNP_12_ (A_1_A_2_)	SNP_13_ (A_2_A_2_)	SNP_21_ (A_1_A_1_)	SNP_22_ (A_1_A_2_)	SNP_23_ (A_2_A_2_)	Class
SAMPLE1	1	0	0	1	0	0	+1
SAMPLE2	0	0	1	0	1	0	-1
SAMPLE3	0	1	0	0	0	1	-1

It should be noted that other methods exist for encoding genetic profiles in numerical format, i.e. by introducing different numbers of variables in the transformed dataset [18,19,21,24]. The number of variables obtained is critical for the choice of the methodology to be used, as it may emphasize or overlook the dominant/recessive characteristics of the alleles to be analyzed [20,22]. Even though the selected code could turn out to be quite cumbersome, such encoding preserves all the information harboured by the original data. In addition, it is worth to note that the adopted data encoding allows a simple and direct treatment of missing values. Let us suppose that the information about the genotype of the generic *SNP*_*j *_is missing, the three binary variables *SNP*_*jk *_will all be assigned the value 0. This kind of representation adeguately models the absence of information and does not require complex data imputation methodologies.

### Datasets

We used several datasets in order to demonstrate the validity of our similarity measure. In particular, many simulated datasets were generated in order to mimic data collection as faithfully as possible in order to study phenotypes influenced by genetic factors.

In a second approach, we used a real data set obtained by genotyping a group of subjects affected by Sporadic Colorectal Cancer (cases) and a comparabale number of healthy subjects (controls). In all the cases, to avoid biased estimates due to the phenotypic selection, we estimated the allele frequencies from the control group (class -1) of each dataset.

#### a. Simulated dataset

We assumed the existence of a generic population, where one percent (1%) of subjects is affected by a specific phenotype. The subjects with the phenotype under study are called cases, while the remaining subjects are controls. We further assumed that all subjects of the population were genotyped for 20 different SNPs. Among the 20 SNPs, 5 were assumed to be informative with respect to the phenotype, while the remaining were not influencing (they can be considered as noise).

For all the informative SNPs, we assigned allele *A*_1 _with a frequency *p*_-1 _for the control subjects. Thus, the other allele *A*_2 _will be forced to have a frequency *q*_-1 _= 1 - *p*_-1_, while the genotype frequencies will be determined by the Hardy-Weinberg law Table 3.

**Table 3:** Genotypic frequencies as determined by Hardy-Weinberg law given two alleles A_1 _(with a frequency of *p*) and A_2 _(with a frequency of *q*).

**Genotype**	**Frequency**
*A*_1_*A*_1_	p^2^
*A*_1_*A*_2_	2 pq
*A*_2_*A*_2_	q^2^

For the cases, the *A*_1 _alleles of the informative SNPs had a frequency equal to *p*_1 _= *r*·*p*_-1 _where *r *is a real number greater than one. That is, the allele *A*_1 _of each informative SNPs is more common in the subset of the population affected by the phenotype than in the control subset. In this way we created a relationship between the informative SNPs and the phenotype itself.

In order to vary the strength of the relationship between the informative SNPs and the phenotype, we adopted different rules for the generation of *r*:

1. *r *values set to 1.5;

2. *r *values set to 2;

3. *r *values set to 2.5;

4. *r *values set to 3;

5. *r *values randomly chosen in the interval [1.5, 3].

In cases 1–4 the strength of the relationship grows, while the fifth case ensures there is a variable strength of relationship for each informative SNP.

Regarding the 15 uninformative SNPs, we set that they are not related to the presence of phenotype, by simply forcing the genotypes frequencies at thirty three percent both for the cases and for the controls.

At this point, we generated several populations by varying the *p*_-1 _frequency and the rule for the setting of the *r *values. In particular, the *p*_-1 _frequency ranged from 0.005 to 0.10 with a step of 0.005, and from 0.11 to 0.33 with a step of 0.01. It should be noted that the *p*_-1 _frequency is low, the informative SNPs have a rare allele that influence the presence of the phenotype, while when the *p*_-1 _frequency is high, the informative SNPs do not have any rare genotype. In such a way we can test the validity of our kernel either in situations where we expect a better performance (rare alleles), or in those situations where the HWk and the linear kernel should not show significant differences.

In synthesis, for each value of *r*, we generated a total of 43 populations (corresponding to the 43 values of *p*_-1_); each population was composed by 30000 subjects, and then approximately 300 subjects were cases. From each population we extracted only one dataset by selecting all the cases and an equal number of randomly chosen controls.

Finally, we generated a last set of populations, by introducing a different kind of noise. The new noising SNPs were characterized by having a rare allele, with the same frequency for both cases and control. We replaced 5 of the uninformative SNPs with 5 of the new noising SNPs. In this way we wanted to test whether the HWk performance is influenced by the presence of uninformative SNPs with rare alleles. For this last set of populations, we used the same values of *p*_-1 _frequency that we used for the other populations, but only the fifth rule for the generation of *r *values.

#### b. Dataset on sporadic colorectal cancer

To further test the function of our kernel function we used a dataset which included genotypic data collected in the context of a study aimed at investigating the role of genetic variability of candidate genes in the susceptibility to Sporadic Colorectal Cancer (SCC). A complete list of the genes and of the polymorphisms can be requested from VM.

Genotyping was carried out by APEX technology [25] on a sample of unrelated subjects recruited from the Spanish population which included a group of 377 patients affected by SCC (cases) and a group of 329 healthy subjects representing the general population (controls), matched for age, sex and ethnicity with the cases. A total of 160 informative SNPs linked to 66 genes were used. These genes were involved in the metabolism of dietary carcinogens and xenobiotics, in the DNA repair and in the apoptotic process. Some of the data included in this dataset have previously been analyzed and the results published [26-29].

## Results and discussion

The proposed HWk was obtained by modelling Hardy-Weinberg law into a linear kernel function. In order to introduce the HWk, we will first describe how the similarity measure between two genetic profiles, *X*_1 _and *X*_2 _is computed using the well known linear kernel function *K*_*L*_:

(1)

As we stated in the "Methods" section the indexes *j *and *k *represent, respectively, the *j*^*th *^SNP with the *k*^*th *^genotype. It is clear that the similarity measure computed by the linear kernel merely consists in the sum of SNPs presenting the same genotypes in both genetic profiles X_1 _and X_2_.

In order to model the Hardy-Weinberg law into a linear kernel function, we introduced a weight *w*_*jk *_for each variable *SNP*_*jk*_. The HWk *K*_*HW *_can now be defined as:

(2)

The weights *w*_*jk *_were defined in terms of genotypic frequencies *f*_*jk *_as:



where *f*_*jk *_is defined according to the Hardy-Weinberg law by the frequencies of the alleles it is made of.

The term  encodes the inverse relationship between *f*_*jk *_and *w*_*jk*_; the term  is a regularization term that normalizes the weights of the generic SNP *j *with respect to its highest genotypic frequency. Consequently, *w*_*jk *_assumes the highest values for rare genotypes and the smallest values for common genotypes.

### Experimentation protocol

The main objective of our experimentation protocol was to demonstrate that the incorporation of previous data derived from the Hardy-Weinberg law in a kernel function provides sensible advantages.

In order to achieve this objective, we compared the performance obtained using the HWk embedded in SVM models to results obtainable using the well known linear kernel.

We chose to compare the HWk with the linear kernel because the first can be considered as an extension of the latter. Thus, we attempted to measure the advantage of employing the genotype frequencies calculated according to Hardy-Weinberg equilibrium. Moreover, we had to choose a class of classification function in order to test our similarity measure, since kernel functions are exclusively used embedded in classification/regression algorithms. SVM models seemed to be the most suitable choice, given their wide applicability.

A drawback of SVM algorithms is that SVM require the choice of a regularization parameter, called *C*. This parameter can be thought as a lever for regulating the training phase of SVM models: high *C *values force the SVM training algorithm to be more sensitive to the presence of outliers, while lower *C *values make SVM models more robust but can fail in detecting the real shape of the data. Machine Learning practitioners usually adopt the cross-validation technique in order to determine the optimal values of *C*. Cross-validation consists in splitting the dataset in *n *folds and repetitevily hold out one fold while the remaining ones are used to fit the model. The *n *models are then tested on the respective hold – out fold. In this way it is possible to obtain an almost unbiased estimation of SVM model performances. However, cross-validation performance estimation strongly depends on the dataset splitting. Thus, we adopted a *repeated cross-validation technique*, by repeating the whole cross-validation procedure multiple times and then averaging the results. Schematically, our experimentation protocol can be described as follows:

1. Repeat *n *times:

a. Split the dataset D in *k *different folds.

b. For each *C *value:

i. Perform a whole cross-validation on the *k *different folds.

2. For each *C *value, average the *n *performance values.

3. Select the best values of *C*.

Please note that we average *n *cross-validated performances for each values of *C*, and then we selected the best *C *value. Therefore our choice is far away more robust than using a single cross-validation. The whole experimental protocol was repeated once for each kernel function.

A key point of each model evaluation procedure is the choice of an appropriate metric; for our experimentation protocol we adopted the Area Under the Curve (AUC) measure. AUC metric is a widely used performance metric in the field of Machine Learning [30], since AUC exhibits a number of advantages with respect to other simpler performance measures, including independence from the decision threshold, invariance with respect to a priori class probabilities, and it gives low scores to both random and "one class only" classifiers.

The protocol was applied to both the simulated and the SCC datasets. However, the SCC dataset was treated with some additional pre-processing steps. In fact, we preliminarly eliminated the SNPs that did not comply to Hardy-Weinberg equilibrium. Then, we further reduced the number of variables of the real dataset, by applying an univariate feature selection protocol. In particular, we selected all the variables that were significatively correlated to the class attribute from a *χ*^2 ^test. Only the variables with p-values lower than a threshold *T *were selected. We used three significance thresholds, namely 0.1, 0.15 and 0.2, and thus we constructed three reduced datasets from the original one. The elimination of uncorrelated features was necessary in order to eliminate possible noise. Actually, a far more sophisticated feature selection procedure could be employed, but we decided to use the simple univariate selection procedure in order to maintain the focus of our experimentation on the differences between the linear and the HWk.

The experiments were run using a computer program implemented in MATLAB; the script was interfaced with the software package LIBSVM [31] for the training of the SVM models. The code is freely available from  or upon request from the corresponding author.

### Computational results

**Figure 1:**
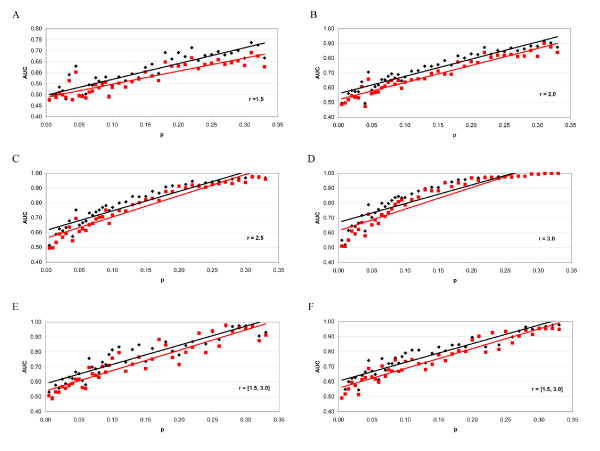
**Results obtained on the simulated datasets**. Each set of figures refers to different rules for the generation of the *r *parameter (r = 1.5 (a), r = 2.0 (b), r = 2.5 (c), r = 3.0 (d), r in [1.5–3.0] (e), r in [1.5–3.0] with "low frequency uninformative" SNPs (f)); the horizontal axis reports the frequency *p *of the allele A1 of the relevant SNP, while the vertical axis reports the best cross validated AUC values obtained with HW (black) and linear (red) kernels.

Figure 1 summarizes the results obtained on the simulated datasets. Each figures refers to different rules for the generation of the *r *parameter; the horizontal axis reports the *p*_-1 _frequency, while the vertical axis reports the best cross-validated AUC values obtained with HW and linear kernels.

Figure 1 shows that the HWk always obtains greater or equal AUC values than those obtained by the linear kernel. The pattern followed by the results is independent of the procedure for the generation of *r*; also the datasets generated with a different type of noisy SNPs follow the same behaviour of the other datasets.

We observed an evident linear trend in each figure: the performances of both HW and linear kernels tend to growth as *p*_-1 _increases. We can explain this trend by looking at the odds ratio (OR) between cases and controls with respect to a single informative SNP. This OR can be formulated in terms of *p*_-1_, *p*_1 _and *r*:

(3)

In (3), as *p*_-1 _increases, the numerator decreases, but the denominator diminishes faster, since *r *is greater than one. Then the OR grows with *p*_-1_, and the SNP becomes more informative.

Lastly, we noted that the performances of the HWk kernel follow a characteristic trend also with respect to the *C *parameter of the SVM classifier. In fact, we observed that the best AUC values were always obtained with small *C *values, while for higher *C *values the performances of the HW and linear kernels tended to the same value.

**Figure 2:**
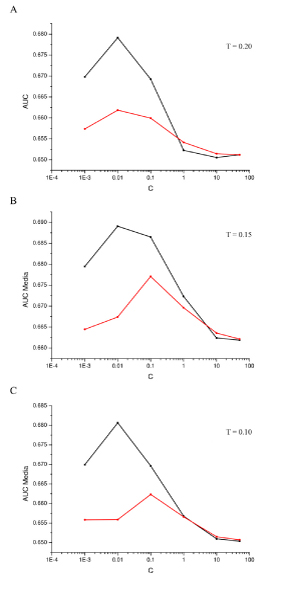
**Mean values of the AUC performances computed on the five repeated cross-validations obtained using a SVM classifier with either HWk (black) and the linear kernel (red) with the SCC dataset**. The results refer to a threshold *T *for the univariate feature selection procedure of respectively 0.20 (a), 0.15 (b) and 0.10 (c).

The results using the SCC dataset confirm the characteristics of the HWk already pointed out with the experiments on simulated datasets. Figures 2a, 2b and 2c report the results for the SCC dataset with a threshold *T *for the univariate feature selection procedure of respectively 0.2, 0.15 and 0.10. The results for each repetition of the cross-validation procedure are reported in Additional file 1, Tables S1a S1b and S1c. We can observe that the performances of HWk are better for small values, while the two kernels shows similar results with higher *C *values.

## Conclusion

We set up a new kernel, the HWk, which is specific for handling genetic profiles data and that performs better than the linear kernel. It is worth noting that, the HWk described here represents one of the first attempts aimed at inserting specific-domain knowledge into the definition of a kernel function specifically devised for the analysis of SNPs in complex phenotypes. The results obtained from the simulated data, as well as the results on the SCC dataset, pointed out that the HWk always obtains better performance than the linear kernel, especially with small *C *values. This characteristic can be explained by noting that the HWk essentially multiplies the variables corresponding to rare genotypes for positive factors. In fact, by multiplying a relevant dimension (i.e. a dimension that gives a strong contribution to the separation of the two classes) it is possible to obtain SVM models which are more robust, because of a larger separation margin. A wide separation margin ensures better generalization capabilities, that is a higher probability of correctly classifying new instances [32]. On the other hand, multiplying an irrelevant feature leads to the same result of the absence of stretching.

In other words, the performance of HWk is always favourable with respect to linear kernel, but such a difference in performance is maximum when relatively rare genotypes (about 10%) are crucial for distinguishing between cases and controls, that is to say they have a role in the susceptibility to the trait analyzed. This is a very important and useful feature, as most of the current statistical tools loose most of their statistical power when rare genotypes are involved in the susceptibility to the trait under study. Thus, HWk may represent a valuable tool for the case-control studies carried out with high-throughput genotyping. Finally, it might be worth noting that in most of the human complex traits, the genetic component account for 10–50% of the individual risk. That is, in most cases the possibility to estimate the individual risk on the basis of genetic factors is quite low [33]. Thus, the set up of a statistical tool that consistently improves the probability of correct classification of about 5% may represent an important step forward in this field.

## List of abbreviations used

SVM: Support Vector Machine; SNP: Single Nucleotide Polymorphism; DNA: Deoxyribonucleic Acid; PCR: Polymerase Chain Reaction; APEX: Arrayed Primer Extension; SCC: Sporadic Colorectal Cancer; HWk: Hardy-Weinberg kernel; AUC: Area Under the Curve.

## Competing interests

The authors declare that they have no competing interests.

## Authors' contributions

VL and AM carried out all the computational experiments. FDC, SL, VM provided the molecular data of the samples included in the dataset. DC, GR and GP conceived the study, and participated in its design and coordination. The Ms was initially drafted by GP and then finalized with VL, AM, FDC and GR. All the authors read and approved the final manuscript.

## Supplementary Material

Additional file 1 The results for each repetition of the cross-validation procedure. Click here for file
