# Supplementary material for: A novel similarity-measure for the analysis of genetic data in complex phenotypes
Source: BMC Bioinformatics. 2009 Jun 16;10(Suppl 6):S24. doi: 10.1186/1471-2105-10-S6-S24 (PMC2697648; doi:10.1186/1471-2105-10-S6-S24)
Supplement: Additional file 1 — The results for each repetition of the cross-validation procedure. [file 1471-2105-10-S6-S24-S1.doc]

**Table S1. Supplementary Material.** Results of the repeated cross-validation procedure on the SCC dataset. Each row refers to one of the five different cross-validations, while each column refers to a different value of *C*. Each table reports both HWk and linear kernel AUC performances. For each *C* value the  *t*-test was performed to compare the AUC performances obtained with the two kernel functions. The results refer to a threshold *T* for the univariate feature selection procedure of respectively 0.2 (a), 0.15 (b) and 0.10 (c).

a. Chi-square with univariare feature selection threshold *T* <0.20

|  | **HW Kernel** | | | | | |
| --- | --- | --- | --- | --- | --- | --- |
|  | *C=0.001* | *C=0.01* | *C=0.1* | *C=1* | *C=10* | *C=50* |
| 1 | 0.672 | 0.683 | 0.670 | 0.656 | 0.652 | 0.653 |
| 2 | 0.675 | 0.684 | 0.684 | 0.665 | 0.661 | 0.663 |
| 3 | 0.662 | 0.669 | 0.656 | 0.644 | 0.640 | 0.640 |
| 4 | 0.669 | 0.676 | 0.657 | 0.641 | 0.646 | 0.645 |
| 5 | 0.671 | 0.683 | 0.679 | 0.655 | 0.654 | 0.654 |
| Mean | 0.670 | 0.679 | 0.669 | 0.652 | 0.651 | 0.651 |
|  |  |  |  |  |  |  |
|  | **Linear Kernel** | | | | | |
|  | *C=0.001* | *C=0.01* | *C=0.1* | *C=1* | *C=10* | *C=50* |
| 1 | 0.666 | 0.671 | 0.667 | 0.659 | 0.653 | 0.653 |
| 2 | 0.658 | 0.664 | 0.664 | 0.669 | 0.660 | 0.663 |
| 3 | 0.647 | 0.653 | 0.652 | 0.641 | 0.642 | 0.640 |
| 4 | 0.650 | 0.656 | 0.652 | 0.644 | 0.645 | 0.645 |
| 5 | 0.665 | 0.666 | 0.665 | 0.659 | 0.658 | 0.655 |
| Mean | 0.657 | 0.662 | 0.660 | 0.654 | 0.651 | 0.651 |
|  |  |  |  |  |  |  |
| t-test | 0.0114 | 0.0021 | 0.0949 | 0.6074 | 0.5677 | 0.4998 |

b: Chi-square with univariare feature selection threshold *T* <0.15

|  |  | | | | | |
| --- | --- | --- | --- | --- | --- | --- |
|  | **HW Kernel** | | | | | |
|  | *C=0.001* | *C=0.01* | *C=0.1* | *C=1* | *C=10* | *C=50* |
| 1 | 0.675 | 0.691 | 0.691 | 0.669 | 0.654 | 0.652 |
| 2 | 0.685 | 0.696 | 0.700 | 0.689 | 0.681 | 0.680 |
| 3 | 0.676 | 0.678 | 0.674 | 0.660 | 0.649 | 0.649 |
| 4 | 0.679 | 0.684 | 0.681 | 0.668 | 0.661 | 0.661 |
| 5 | 0.683 | 0.696 | 0.688 | 0.676 | 0.667 | 0.668 |
| Mean | 0.679 | 0.689 | 0.687 | 0.672 | 0.662 | 0.662 |
|  |  |  |  |  |  |  |
|  | **Linear Kernel** | | | | | |
|  | *C=0.001* | *C=0.01* | *C=0.1* | *C=1* | *C=10* | *C=50* |
| 1 | 0.672 | 0.675 | 0.681 | 0.668 | 0.656 | 0.653 |
| 2 | 0.665 | 0.667 | 0.688 | 0.688 | 0.681 | 0.679 |
| 3 | 0.657 | 0.662 | 0.661 | 0.656 | 0.651 | 0.649 |
| 4 | 0.659 | 0.664 | 0.673 | 0.668 | 0.662 | 0.662 |
| 5 | 0.670 | 0.669 | 0.682 | 0.669 | 0.668 | 0.668 |
| Mean | 0.664 | 0.667 | 0.677 | 0.670 | 0.664 | 0.662 |
|  |  |  |  |  |  |  |
| t-test | 0.0015 | 3.95E-04 | 0.0917 | 0.357 | 0.5594 | 0.5134 |

c: Chi-square with univariare feature selection threshold *T* <0.10

|  |  | | | | | |
| --- | --- | --- | --- | --- | --- | --- |
|  | **HW Kernel** | | | | | |
|  | *C=0.001* | *C=0.01* | *C=0.1* | *C=1* | *C=10* | *C=50* |
| 1 | 0.672 | 0.689 | 0.681 | 0.667 | 0.658 | 0.658 |
| 2 | 0.670 | 0.670 | 0.657 | 0.641 | 0.635 | 0.634 |
| 3 | 0.675 | 0.677 | 0.663 | 0.648 | 0.641 | 0.641 |
| 4 | 0.672 | 0.687 | 0.678 | 0.665 | 0.660 | 0.659 |
| 5 | 0.661 | 0.680 | 0.669 | 0.662 | 0.661 | 0.660 |
| Mean | 0.670 | 0.681 | 0.670 | 0.657 | 0.651 | 0.650 |
|  |  |  |  |  |  |  |
|  |  |  |  |  |  |  |
|  | **Linear Kernel** | | | | | |
|  | *C=0.001* | *C=0.01* | *C=0.1* | *C=1* | *C=10* | *C=50* |
| 1 | 0.662 | 0.663 | 0.673 | 0.669 | 0.658 | 0.659 |
| 2 | 0.650 | 0.651 | 0.651 | 0.641 | 0.635 | 0.634 |
| 3 | 0.653 | 0.656 | 0.663 | 0.648 | 0.642 | 0.641 |
| 4 | 0.664 | 0.662 | 0.664 | 0.664 | 0.662 | 0.660 |
| 5 | 0.650 | 0.647 | 0.661 | 0.661 | 0.662 | 0.661 |
| Mean | 0.656 | 0.656 | 0.662 | 0.657 | 0.652 | 0.651 |
|  |  |  |  |  |  |  |
|  |  |  |  |  |  |  |
| t-test | 0.0026 | 2.84E-04 | 0.1136 | 0.4905 | 0.5269 | 0.5167 |
